# Supplementary material for: Influence of dietary composition on the nutritional profile and feed conversion efficiency of Tenebrio molitor
Source: PLoS One. 2025 Jul 10;20(7):e0325262. doi: 10.1371/journal.pone.0325262 (PMC12244549; doi:10.1371/journal.pone.0325262)
Supplement: S1 File — (DOCX) [file pone.0325262.s001.docx]

**
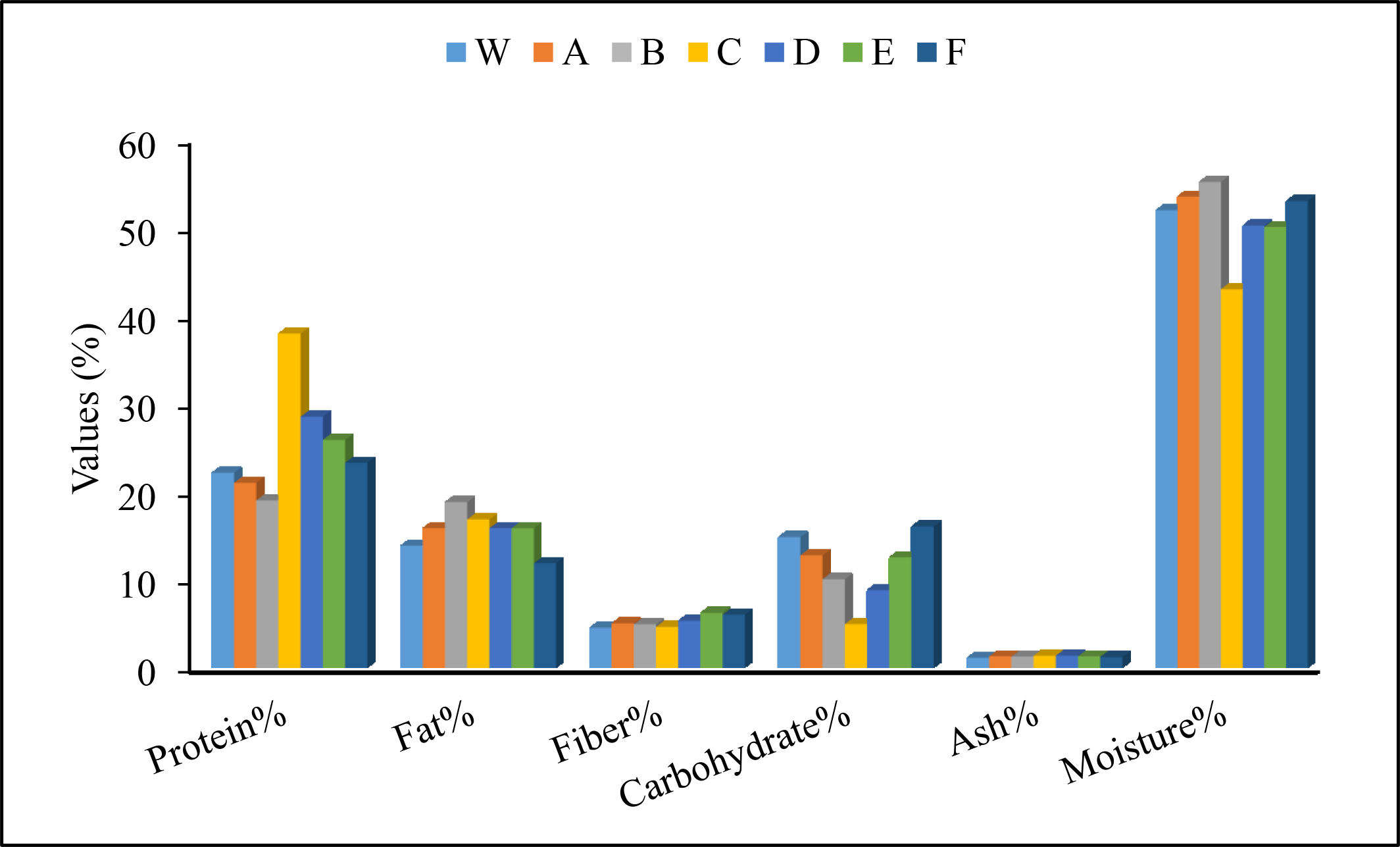
**

**Fig S1.** Comparative chart of the nutritional profile of *Tenebrio molitor* larvae in seven experimental diets A (50% wheat bran + 50% barley bran), B (75% wheat bran + 25% barley bran), C (50% chickpea bran + 50% wheat bran), D (25% chickpea bran + 75% wheat bran), E (50% corn bran + 50% wheat bran), F (25% corn bran + 75% wheat bran), and W (100% wheat bran).


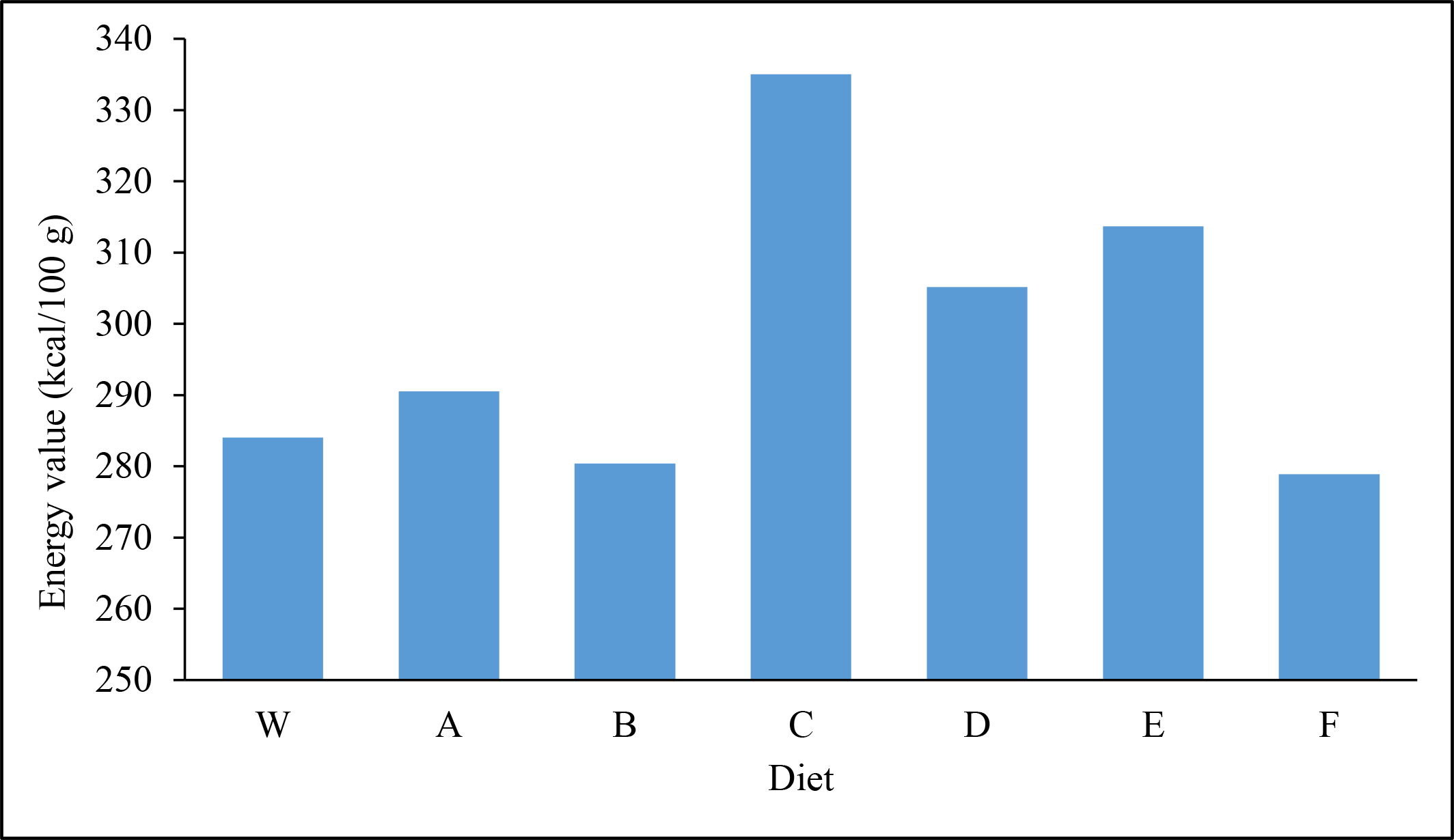


**Fig S2.** Comparative chart of the Energy value (kcal/100 g) of *Tenebrio molitor* larvae in seven experimental diets. A (50% wheat bran + 50% barley bran), B (75% wheat bran + 25% barley bran), C (50% chickpea bran + 50% wheat bran), D (25% chickpea bran + 75% wheat bran), E (50% corn bran + 50% wheat bran), F (25% corn bran + 75% wheat bran), and W (100% wheat bran).
