# Supplementary material for: Influence of dietary composition on the nutritional profile and feed conversion efficiency of Tenebrio molitor
Source: PLoS One. 2025 Jul 10;20(7):e0325262. doi: 10.1371/journal.pone.0325262 (PMC12244549; doi:10.1371/journal.pone.0325262)
Supplement: Attachment — Submitted filename: Response to Reviewers.docx [file pone.0325262.s004.docx]

**Response to Reviewer Comments**

We sincerely appreciate the reviewers' insightful and constructive comments, which have greatly contributed to improving the quality and clarity of our manuscript. Addressing these comments has allowed us to refine our study and better highlight its significance. Below, we provide detailed responses to each of the reviewers' comments, outlining the specific revisions made to the manuscript. We are grateful for the time and effort the reviewers have dedicated to evaluating our work and hope that the revised version meets their expectations. Thank you for your valuable feedback and consideration."

**Reviewer #1:**

Comment 1: It is unclear how many mealworms were used per treatment and whether replicates were biological or technical.
The manuscript states that three replicates per diet were used, but this may not be sufficient for robust statistical comparisons.

Response Thank you for raising this important point. To clarify, each replicate consisted of fifty larvae, which were randomly assigned to a plastic container (22 × 28 × 15 cm) equipped with aeration slits on the sides. The three replicates per diet were biological replicates, meaning they represented independent groups of larvae raised under the same dietary conditions. We appreciate your feedback and have revised the manuscript to include these clarifications."

Comment 2: The study does not report mycotoxin screening or contamination control in dietary ingredients.
Given that bran-based diets can be prone to fungal or bacterial contamination, this omission is a potential flaw affecting validity.

Response Prior to use, the ingredients (wheat bran and other grains) were mixed, homogenized, and stored at 4°C for 24 hours to reduce fungal and microbial contamination in the mealworm medium.

Refrences: Li L, Zhao Z, Liu H. Feasibility of feeding yellow mealworm (Tenebrio molitor L.) in bioregenerative life support systems as a source of animal protein for humans. Acta Astronautica. 2013;92(1):103-9.

Comment 3: While protein content is analyzed, no detailed amino acid profile is provided, which is crucial for evaluating the nutritional quality of mealworms.

Response We appreciate this valuable observation. As this study is part of an MSc thesis, the scope was focused on analyzing the protein content of mealworms. However, we fully recognize the importance of evaluating the amino acid profile to comprehensively assess nutritional quality. Future research will expand on these findings by investigating the detailed amino acid composition of mealworms, as well as exploring potential heavy metal accumulation.

Comment 4: The manuscript states that the study was approved by the Razi University Ethics Committee, but the specific ethical considerations for insect research are not discussed. More details on ethical handling and humane euthanasia methods for mealworms should be included.

Response Thank you for comment. We have now included additional details regarding the ethical handling and humane euthanasia methods for mealworms in the revised manuscript. The mealworms underwent a 24-hour starvation period, during which their feed was removed. This practice is commonly employed to empty their gastrointestinal tracts, reducing microbial load and enhancing safety for human consumption. After harvest, the larvae were thoroughly rinsed with tap water to remove any residual debris. For humane euthanasia, the larvae were frozen at -20°C. Freezing at this temperature is widely recognized as an ethical method for euthanizing insects, as it minimizes pain and stress by gradually slowing their metabolic rate and physical activity. This approach is considered superior to alternative methods, such as boiling or crushing, and aligns with international research guidelines and standard practices in the edible insect industry (van Broekhoven et al., 2015).

Reference:

van Broekhoven S, Oonincx DGAB, van Huis A, van Loon JJA. Growth performance and feed conversion efficiency of three edible mealworm species (Coleoptera: Tenebrionidae) on diets composed of organic by-products. Journal of Insect Physiology. 2015;73:1-10.

Comment 6: The conclusion claims broad applicability of the findings to mealworm farming, but these claims lack sufficient evidence from large-scale trials. More discussion on real-world feasibility and potential economic implications of using chickpea bran in commercial mealworm farming is needed.

Response Mealworms (*Tenebrio molitor* larvae) are increasingly recognized as a sustainable protein source for both animal feed and human nutrition. Traditionally, wheat bran has been the primary dry food used to rear these insects. However, alternative bran sources, such as barley, corn, and chickpea, may offer unique nutritional profiles that could impact the larvae's growth performance, overall nutritional value, and nutrient absorption. Given the limited research on these alternative substrates, this study hypothesizes that incorporating barley, corn, and chickpea bran into the diet of *T. moli*tor larvae will result in significant differences in their growth rates, nutritional composition, and nutrient absorption efficiency compared to a standard 100% wheat bran diet. Additionally, it is expected that specific combinations or proportions of these alternative brans may optimize the larvae's performance and nutritional outcomes.

Comment 7: More discussion on real-world feasibility and potential economic implications of using chickpea bran in commercial mealworm farming is needed.

Response Thank you for this valuable suggestion. While our findings indicate that chickpea bran shows promise as a dietary substrate for mealworms, it is important to emphasize that these results were obtained under controlled experimental conditions. To fully assess the real-world feasibility and economic viability of incorporating chickpea bran into commercial mealworm farming, additional large-scale trials are necessary.

Comment 8: The discussion cites relevant literature, but comparisons with previous studies are weak.
It is unclear how the results align or contradict existing research on insect nutrition. A more systematic comparison with past studies is needed to establish the novelty of the work.

Response Thank you for this insightful comment. We acknowledge that the discussion could benefit from a more systematic comparison with existing literature to better contextualize our findings. In the revised manuscript, we have strengthened the discussion by explicitly comparing our results with those of previous studies on insect nutrition.

Comment 9: The study evaluates mineral content, but it does not consider possible heavy metal accumulation in larvae, which is a critical concern for human and animal consumption.
A brief discussion of food safety risks would improve the manuscript.

Response We appreciate this important observation. While the current study focused on evaluating the mineral content of mealworms, we recognize that assessing heavy metal accumulation is crucial for ensuring the safety of mealworms as a food source for humans and animals. We emphasize that future research should incorporate a thorough analysis of heavy metal levels in mealworms reared on various diets, including chickpea bran, to address this critical gap.

Comment 10: The manuscript contains several grammatical errors and awkward phrasing that reduce readability.
Example:
"The results showed that diets with 50% chickpea bran and 50% barley bran had the highest and lowest protein content in larvae."
Revision: "Diets containing 50% chickpea bran produced larvae with the highest protein content, while those with 50% barley bran yielded the lowest."

Response Thank you for your feedback. We have carefully reviewed the manuscript and made extensive revisions to correct grammatical errors and improve awkward phrasing. For instance, the sentence you highlighted has been revised as follows: 'Diets containing 50% chickpea bran produced larvae with the highest protein content, while those with 50% barley bran yielded the lowest.' These changes, along with similar revisions throughout the manuscript, have significantly enhanced clarity, readability, and overall flow. We appreciate your attention to detail and hope the revised version meets your expectations."

Comment 11: Some figures (e.g., Fig. 1 and Fig. 3) lack clear labeling and legends.
Tables do not adequately summarize key statistical findings (e.g., missing p-values).

Response Thank you for your valuable feedback. According to one of the reviewers, Figures 1 and 3 have been moved to the supplementary section. In the revised manuscript, we have updated the figures to include clearer labels, legends, and annotations to ensure they are more intuitive and informative. Additionally, we have revised the table 2 to include all relevant statistical findings, such as p-values, to provide a comprehensive summary of the data.

Comment 12: Use and cite the following studies to improve the structure and content of your study:
-A review of cultural aspects and barriers to the consumption of edible insects
-First Report of Hermetia Illucens (Linnaeus, 1758), Black Soldier Fly (Diptera, Stratiomyidae) from Iran
-The Effects of Curcumin Supplementation on Body Weight, Body Mass Index, and Waist Circumference in Patients with Type 2 Diabetes: A Systematic Review and Meta-Analysis of Randomized Controlled Trials
Response Thank you for your suggestions. we have cited the study on the cultural aspects and barriers to the consumption of edible insects.

**Reviewer #2:**

 I have found the manuscript important and worthy of consideration. This study provides valuable insights on “Influence of dietary composition on the nutritional profile and feed conversion efficiency of Tenebrio molitor”. However, it requires minor revisions before the editor makes a final decision.

Comment 1. The abstract is quite long. You can consider making it more concise. Please clarify the practical applications of the findings, like implications for large-scale production.

Response Thank you for your feedback. We have revised the abstract to make it more concise while retaining the key points of the study. Additionally, we have clarified the practical applications of the findings, particularly their implications for large-scale mealworm production.

Comment 2. Could you discuss in the introduction why this study is necessary compared to existing research?

Response Thank you for your valuable suggestion. In the revised introduction, we have emphasized the necessity of this study by highlighting gaps in existing research. Specifically, while previous studies have explored various feed formulations for mealworms, there is limited research on the use of cereal bran, such as chickpea bran, as a sustainable and cost-effective dietary substrate.

Comment 3. In the method section, could you briefly explain why ANOVA and Tukey’s test were chosen?

Response Thank you for your comment. ANOVA was chosen for its ability to compare means across multiple groups, allowing us to assess the overall effects of different bran diets on mealworm growth and nutrition. Tukey’s test was selected post-ANOVA for its effectiveness in identifying specific group differences while controlling for Type I error, ensuring robust and reliable comparisons among the bran treatments.

Comment 4. One suggestion for language and formatting. While reading, I have found some sentences are overly complex, simplify those for readability.

Response Thank you for your suggestion. We have revised the manuscript to simplify overly complex sentences, improving readability without compromising scientific accuracy.

**Reviewer #3:**

Introduction
Comment 1: The research gap is not strongly justified. The introduction should explicitly state what is missing in current knowledge and why this study is needed.

Response Thank you for your feedback. In the introduction, I have now emphasized the necessity of this study by highlighting gaps in existing research, particularly the limited exploration of cereal bran's role in optimizing feed formulations for mealworm production.

Comment 2 The introduction should mention any previous studies that have examined similar dietary influences on mealworms and explain how this study builds upon or differs from them.

Response Previous studies have primarily focused on wheat bran and other single-source diets for mealworms. However, limited research has compared the effects of mixed or alternative bran sources such as barley, corn, and chickpea on mealworm nutrition and growth. By incorporating multiple dietary compositions, this study builds upon existing knowledge by exploring the potential benefits of diverse bran sources and determining optimal feed formulations.

Comment 3 The hypothesis or specific research questions are not explicitly stated. A clear hypothesis would improve focus.

Response Thank you for your feedback. I have now explicitly stated the hypothesis in the text.

Methodology
Comment 4 The study lacks a clear justification for the sample size. How was the number of mealworms per treatment determined? Was there a power analysis?

Response Thank you for raising this important point. The sample size of mealworms per treatment was determined based on preliminary studies and existing literature that suggest sufficient replication is needed to achieve reliable results. Each replicate contained fifty larvae, randomly transferred to a plastic container (22 × 28 × 15 cm) with aeration slits on the sides.

Refrences: Van Broekhoven, S., Oonincx, D. G., Van Huis, A., & Van Loon, J. J. (2015). Growth performance and feed conversion efficiency of three edible mealworm species (Coleoptera: Tenebrionidae) on diets composed of organic by-products. Journal of insect physiology, 73, 1-10.‏

Comment 5 The experiment lasted 12 weeks, but there is no discussion on whether this timeframe is sufficient to assess long-term dietary effects.

Response Thank you for your comment. The 12-week experimental duration was chosen based on the larval life cycle of *T. molitor*, which typically spans about three months, and aligns with established literature demonstrating that this timeframe is sufficient to evaluate growth and developmental outcomes. Additionally, prior studies have shown that initiating growth assessments four weeks into the larval stage provides reliable insights into dietary impacts. Thus, the 12-week period is appropriate for capturing significant dietary effects on growth performance and nutritional composition.

Refrences:

van Broekhoven S, Oonincx DGAB, van Huis A, van Loon JJA. Growth performance and feed conversion efficiency of three edible mealworm species (Coleoptera: Tenebrionidae) on diets composed of organic by-products. Journal of Insect Physiology. 2015;73:1-10.‏

Bjørge JD, Overgaard J, Malte H, Gianotten N, Heckmann L-H. Role of temperature on growth and metabolic rate in the tenebrionid beetles Alphitobius diaperinus and Tenebrio molitor. Journal of Insect Physiology. 2018;107:89-96.

Comment 6 Potential biases: Since mealworm growth can be influenced by factors such as rearing density, genetic variability, and microclimate conditions, were these controlled?

Response Thank you for your comment. To minimize potential biases, we controlled for key influencing factors: rearing density was standardized across all treatments, mealworms were sourced from a genetically homogeneous population, and microclimate conditions (temperature, humidity, and light) were maintained consistently throughout the experiment.

Comment 7 The moisture addition (carrots) could introduce variation. Was this accounted for in data interpretation?

Response Thank you for your comment. The moisture content from carrots was carefully monitored and standardized across all treatments to minimize variability. For moisture, 2 grams of fresh carrot was added twice a week, and old carrot pieces were removed. Any uneaten carrot was dried at 100°C until reaching a constant weight. The dried weight was compared to the dry weight of a carrot piece with the same initial fresh weight, ensuring accurate moisture content adjustments in the analysis.

Results
Comment 8 Some results lack clear interpretation—for example, what biological mechanisms might explain why diet C had the highest conversion efficiency?

Response Diet C, which contained chickpea-based ingredients, likely provided superior energy sources for mealworms. Chickpea bran has a high protein and fiber content, enhancing nutrient absorption and metabolic efficiency, leading to better feed conversion rates.

Comment 9 The statistical significance of certain comparisons is not always explained in a biologically meaningful way.

Response Thank you for your valuable comment. We agree that while statistical significance indicates whether a result is likely due to chance, it does not always reflect biological relevance. In the revised manuscript, we have expanded the discussion to provide a more biologically meaningful interpretation of the findings. For instance, variations in feed conversion efficiency and growth rates are linked to specific dietary compositions and their impacts on digestion and nutrient uptake. By connecting these statistical outcomes to underlying biological mechanisms, we aim to offer a clearer understanding of how different diets influence mealworm performance.

Comment 10 Figures lack sufficient labeling—ensure all legends and axis labels are self-explanatory.

Response All figures have been revised to include clear legends and axis labels. The captions of the figures are designed to provide detailed explanations. According to one of the reviewers, Figures 1 and 3 have been moved to the supplementary section.

**Discussion**

Comment 11 The discussion sometimes repeats results rather than interpreting them in-depth.

Response Thank you for your comment. In accordance with the journal's format, the results and discussion sections have been combined to better illustrate the impact of the findings and improve their clarity. In this revised version, we have taken care to minimize repetition of the results and instead focus on providing a deeper interpretation of the data.

Comment 12 The implications for real-world applications (e.g., poultry feed formulation, industrial insect farming) could be expanded.

Response The findings have direct implications for poultry feed formulation and large-scale mealworm farming. Optimizing bran composition in mealworm diets can enhance feed quality, reduce costs, and improve sustainability in industrial insect production.

Comment 13 It lacks a "Limitations and Future Research" section, which is important for acknowledging study constraints.

Response Thank you for this valuable feedback. A dedicated 'Limitations and Future Research' section has now been included in the revised manuscript.

**Reviewer #4:**

Comment1 The statistics are missing. You must add a statistic model to your results.

Response Thank you for pointing this out. The results are presented as Mean ± Standard Deviation (SD). Statistical analyses were performed using one-way ANOVA, followed by Tukey’s post-hoc test for between-group comparisons, with a significance level set at p < 0.05.

Comment 2 The results must be separated from the discussion because it is better to read you text.

Response In line with the journal's format, the results and discussion sections have been merged to better illustrate the impact of the findings and enhance their clarity.

Comment 3 The figures must be more readable.

Response All figures have been revised to include clear legends and axis labels. The captions of the figures are designed to provide detailed explanations. According to one of the reviewers, Figures 1 and 3 have been moved to the supplementary section.

Comment 4 You don’t Response at the discussion why this research is important

Response We have now included the importance and necessity of this research. Thank you for your valuable feedback. We have now added a section in the introduction to emphasize the importance and necessity of this research. Specifically, this study addresses critical gaps in understanding how alternative and sustainable feed sources, such as chickpea bran, can optimize mealworm growth and nutritional quality. Also, a dedicated 'Limitations and Future Research' section has now been included in the revised manuscript.

**Reviewer #5:**

The article "Influence of dietary composition on the nutritional profile and feed conversion efficiency of Tenebrio molitor" is an original work that brings relevant and detailed aspects about the diet of a species of larvae used in poultry and other animal farming. The methodology of the work is clear and well-conducted. Additionally, the presentation, writing, and discussion of the data are good. I have a few considerations for the authors that could enhance the quality of the work:

Comment 1 In some sections of the Results and Discussion, the authors mention data that are simultaneously shown in figures and tables, such as Figure 1 and Table 1. I believe this redundancy of information is unnecessary. I think Figure 1 could be moved to supplementary material. I suggest revising the tables and figures and keeping only one method of presenting results in the main text.

Response Thank you for your valuable feedback. We agree that avoiding redundancy in presenting data is important for clarity and conciseness. In response to your suggestion, we have revised the manuscript to ensure that each dataset is presented only once in the main text, either as a figure or a table, but not both. Specifically, we have moved Figure 1 to the supplementary material, as it complements the data presented in Table 1. This adjustment streamlines the presentation of results and enhances the readability of the manuscript. We appreciate your thoughtful recommendation and hope these changes address your concern effectively."

Comment: I suggest the authors use only the designations for the diets: A, B, C, etc. At several points, the authors return to mention the composition of the diets already described in the Methods. The authors also cite details contained in the tables, such as averages and percentages, within the text, which makes it tiring to read. I suggest avoiding such repetitions. There are inconsistencies related to the percentages of the diet, for example, between lines 300-301. I suggest reviewing the entire section.

Response Thank you for your constructive feedback. In response to your suggestions, we have made the following revisions to the manuscript: We now consistently use the diet designations (A, B, C, etc.) throughout the text, eliminating unnecessary repetition of diet compositions already detailed in the Methods section. We have streamlined the text to avoid reiterating numerical data that are clearly presented in the tables, ensuring the manuscript is more concise and reader-friendly. The section discussing dietary percentages has been thoroughly reviewed, and any inconsistencies, particularly between lines 300-301, have been corrected.
